# Novel Circulating Biomarkers in Aortic Valve Stenosis

**DOI:** 10.3390/ijms26051902

**Published:** 2025-02-22

**Authors:** Joy Yi-Shan Ong, Sarah Ming Li Tan, Angela S. Koh, William Kong, Ching Hui Sia, Tiong Cheng Yeo, Swee Chye Quek, Kian Keong Poh

**Affiliations:** 1 Department of Cardiology, National University Heart Centre Singapore, Singapore 119074, Singapore; joy.ong@mohh.com.sg (J.Y.-S.O.); sarah.tanml@mohh.com.sg (S.M.L.T.);; 2 National Heart Centre Singapore, Singapore 169609, Singapore; 3 DUKE-NUS Medical School, Singapore 169857, Singapore; 4 Yong Loo Lin School of Medicine, National University of Singapore, Singapore 117597, Singapore

**Keywords:** aortic valve stenosis, biomarkers, pathophysiology, disease progression

## Abstract

The underlying pathophysiology of aortic stenosis and factors affecting its clinical progression remain poorly understood. Apart from B-type natriuretic peptide (BNP), novel and emerging biomarkers have been described in association with aortic stenosis, emphasising the potential for these biomarkers to illuminate on yet unknown mechanisms of its pathogenesis. In this review, we aimed to summarise what is known about aortic stenosis biomarkers, highlight the emerging ones, and provide a roadmap for translating these insights into clinical applications. Among the biomarkers studied, lipoprotein(a) [Lp(a)] has emerged as the most promising for risk stratification. Elevated Lp(a) levels are often associated with more rapid aortic stenosis progression. This detrimental effect is attributed to its role in promoting valve calcification. While other emerging biomarkers such as matrix metalloproteinases, monocytes, and metabolites show promises, their specific roles in aortic stenosis pathophysiology remain less clear. This may be due to their relatively recent discovery. Ongoing research aims to elucidate their mechanisms of action.

## 1. Introduction

In aortic stenosis, valve narrowing increases outflow obstruction, leading to left ventricular hypertrophy and remodelling. This then can cause symptoms, such as syncope, dyspnoea, and angina [1,2]. These symptoms can usually be detected by a physical examination, but the diagnosis of aortic stenosis is typically confirmed by echocardiography, based on parameters such as aortic valve area, mean pressure gradient across the valve, and peak jet velocity [3]. Current guidelines advocate for transcatheter aortic valve implantation (TAVI) or surgical aortic valve replacement (SAVR) in patients with symptomatic severe aortic stenosis, as determined by these echocardiographic parameters [3,4]. However, the optimal management strategy for individuals at risk or with asymptomatic severe aortic stenosis remains uncertain.

Recent research has focused on identifying circulating biomarkers to improve the diagnosis. One promising example is B-type natriuretic peptide (BNP). Elevated BNP levels in patients with asymptomatic severe aortic stenosis and preserved left ventricular function predict an increased risk of adverse events; while conversely those with low BNP levels have a lower event rate [5]. This suggests BNP may aid echocardiography in risk stratification. Indeed, BNP has been incorporated into current guidelines [3,4]. However, its clinical utility might be limited by conditions such as renal dysfunction and ageing that can influence its levels [6,7,8,9,10,11,12,13,14]. Therefore, the discovery of additional biomarkers may help to enhance the overall sensitivity of the current aortic stenosis diagnosis.

**Figure 1:**
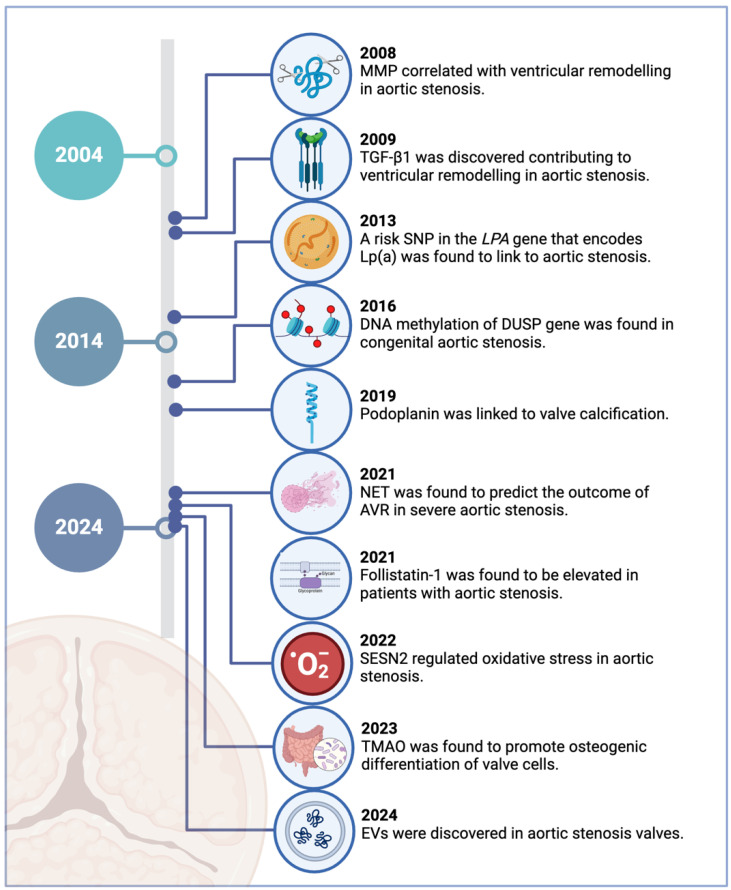
Key milestones in the identification of novel biomarkers for aortic stenosis in the last 20 years. Created in BioRender.com.

This review focuses on recent discoveries of circulating biomarkers for aortic stenosis in the last two decades (Figure 1). Notably, this period has witnessed the emergence of factors previously thought peripheral to cardiovascular conditions, such as gut microbiome-derived metabolites, glycoproteins, and extracellular vesicles. While their clinical utility as a biomarker remains under investigation, these discoveries nevertheless broaden our understanding of the disease’s multifaceted nature. Moreover, we also examine known cardiovascular risk factors, including monocytes and Lp(a), whose roles specifically in aortic stenosis have only been elucidated within this timeframe. By comprehensively analysing these emerging and established factors, we aim to consolidate current understanding of aortic stenosis pathophysiology.

## 2. Pathophysiology of Aortic Stenosis

While once viewed as a passive, age-related process, aortic stenosis is now recognised as an active disease [2]. Its progression can be divided into two phases: initiation and propagation [15]. The initiation phase involves endothelial injury and the subsequent infiltration of Lp(a) and low-density lipoproteins (LDLs) into the valve. Haemodynamic stress likely contributes to endothelial damage [16]. Once infiltrated, they are oxidised by reactive oxygen species, stimulating the extravasation of monocytes and their differentiation into macrophages. This inflammatory cascade leads to foam cell formation and the recruitment of additional immune cells.

The subsequent propagation phase is mainly characterised by fibrosis and calcification. Inflammation-activated vascular interstitial cells contribute to fibrosis by secreting matrix metalloproteinases (MMPs) and adopting a myofibroblastic phenotype [17]. This fibrotic tissue acts as a nidus for calcification, facilitated by inflammation-induced apoptosis of vascular interstitial cells and the release of calcifying microvesicles. Additionally, vascular interstitial cells can drive macrocalcification by transitioning to an osteoblast-like phenotype, promoted by dysregulation of osteogenic mediators [18].

## 3. Challenges in Aortic Stenosis Staging Guidelines

The American College of Cardiology and American Heart Association (ACC/AHA) Heart Valve Disease 2020 Guidelines [3] classify aortic stenosis patients into four stages: at risk, progressive (mild to moderate), asymptomatic severe, and symptomatic severe. This staging is based on valve anatomy, haemodynamics, and symptoms. Valve haemodynamics are assessed using either transthoracic echocardiography or cardiac catheterisation, with aortic valve area (AVA), transaortic maximum velocity (V_max_), and mean pressure gradient (ΔP) being key parameters.

Despite the established guidelines, aortic stenosis staging remains a challenge due to its variable pathophysiology. Several factors, including older age, valve calcification, hypertension, obesity, smoking, hyperlipidaemia, renal insufficiency, and metabolic syndrome, are associated with rapid haemodynamic deterioration [2]. For example, moderate aortic stenosis, even in the absence of overt left ventricular (LV) systolic dysfunction, can be associated with increased risk of accelerated progression, heart failure hospitalisations, and decreased survival [19,20]. While V_max_ and the plasma levels of BNP can provide valuable information about disease stages, their prognostic value is uncertain [21] and current guidelines [3] still do not recommend AVR unless cardiac surgery is required for other indications. Therefore, understanding its pathophysiology through biomarkers could refine or add criteria in the management of aortic stenosis.

## 4. Novel Circulating Biomarkers in Aortic Stenosis Pathogenesis

In the following sections, we will concentrate on factors identified within the past two decades that have not been extensively studied in the context of aortic stenosis. We will exclude well-established markers such as C-reactive protein, BNP, NT-pro-BNP, troponin, gamma-glutamyltransferase, tumour necrosis factor, IgM, calcium–phosphorus products, von Willebrand factor, leptin, adiponectin, homocysteine, endothelin-1, angiotensin II, galectin-3, LDL, asymmetric dimethylarginine, and F-sodium fluoride. It is important to note that the selection of biomarkers discussed in this review was not based on a formal systematic review protocol but rather an approach aimed at highlighting promising and clinically relevant candidates for aortic stenosis diagnosis. We prioritised biomarkers demonstrating clinical relevance through prior investigation in the context of aortic stenosis, emerging evidence suggesting potential clinical utility (even if requiring further validation), and biological plausibility based on their known involvement in the pathophysiology of the disease, such as in processes related to inflammation, calcification, or left ventricular remodelling. Furthermore, we considered the availability of sufficient published data to support a meaningful discussion of each biomarker’s potential role. This selection strategy sought to balance breadth of coverage with depth of analysis, focusing on biomarkers that hold the most promise for improving aortic stenosis care.

### 4.1. Lipoprotein(a)

#### 4.1.1. Conjugated with Oxidised Phospholipids

Lipoprotein(a) [Lp(a)], the key carrier of oxidised phospholipids in the blood circulation, consists of a low-density lipoprotein (LDL) particle bound to apolipoprotein (a), which is encoded by the *LPA* gene [22,23]. Following the discovery of a single nucleotide polymorphism (SNP) in this gene (rs10455872) [24], several studies have consistently supported the association between elevated Lp(a) levels and increased aortic stenosis risk across diverse populations [25,26,27,28].

Elevated Lp(a) levels may contribute to aortic valve calcification by activating the autotaxin-lysophosphatidic acid signalling pathway in the valvular interstitial cells [29,30,31]. Oxidised phospholipids carried on Lp(a) can disrupt the barrier of these cells, allowing Lp(a) to penetrate underneath [32]. Once inside, Lp(a) stimulates autotaxin and lysophosphatidic acid, leading to the expression of pro-inflammatory factors such as interleukin-6 and nuclear factor-κB. This pro-inflammatory environment activates osteogenic genes, such as *RUNX2* and *BMP2*, driving valve calcification [33,34,35,36]. Additionally, Lp(a) contributes to oxidative stress through signalling pathways involving *NOTCH1* activation, further promoting valve calcification [37,38,39,40].

While elevated Lp(a) levels are often associated with faster disease progression in aortic stenosis [41,42,43,44,45], studies have demonstrated a strong association between elevated Lp(a) and the development of subclinical asymptomatic aortic sclerosis, characterised by valve thickening without significant obstruction to blood flow [38,44]. This association, however, may not be as pronounced in patients with severe symptomatic aortic stenosis [46], suggesting a differential impact of Lp(a) on the various stages of the disease.

Consistent with this hypothesis, population-based studies have demonstrated a link between Lp(a) and the early stages of aortic stenosis, particularly in terms of baseline and new-onset valve calcification, but not with stenosis progression [47,48]. Indeed, in patients with mild to moderate aortic stenosis, valve calcification activity is primarily driven by the existing calcium burden, not Lp(a) [49]. These findings suggest that Lp(a) plays a pivotal role in the initiation and early development of the disease, rather than its progression to severe stages. In addition, the limited effectiveness of lipid-lowering interventions in attenuating aortic stenosis progression, especially in moderate to severe stages [50], further supports the notion that Lp(a) may be a more significant risk factor for early progression.

It is noteworthy that the role of Lp(a) may be influenced by what molecules it interacts with. For example, when conjugating with apolipoprotein C-III, Lp(a) is associated with moderate stage rather than early stage involvement [51,52]. We will delve into this further in the next section. Furthermore, the impact of Lp(a) on aortic stenosis may vary across different populations. While elevated Lp(a) levels have been associated with aortic stenosis in white and black individuals, this association has not been observed in Hispanic or Chinese populations [53]. Also, some studies have even suggested a lack of association between Lp(a) and early-stage calcific valve stenosis [54,55]. These contradictory findings emphasise the potentially complex role of Lp(a) in the development of aortic stenosis.

Despite these complexities, the established role of Lp(a) in aortic stenosis warrants further investigation into its potential as a biomarker. Recent drug discoveries [56], including the application of gene silencing technology to regulate Lp(a) expression via its *LPA* gene, have paved the way for promising clinical trials in aortic stenosis patients (Table 1). Lp(a)FRONTIERS CAVS trial (NCT05646381), currently recruiting 502 subjects and anticipated to be completed in January 2029, exemplifies this burgeoning field.

#### 4.1.2. Conjugated with Apolipoprotein C-III

Lp(a) can also bind to apolipoprotein C-III, a small circulating apolipoprotein that regulates plasma triglyceride metabolism. It promotes hypertriglyceridaemia by inhibiting lipoprotein lipase activity, interfering with lipoprotein catabolism, and promoting the assembly of very low density lipoprotein, ultimately increasing the risk of atherosclerosis [59,60,61]. While the atherogenic role of apolipoprotein C-III is primarily mediated through its effects on triglyceride-rich lipoproteins, such as chylomicrons and very low density lipoprotein, its impact on aortic stenosis seems to be particularly associated with Lp(a) [52]. Indeed, apolipoprotein C-III-Lp(a) complexes have been detected in aortic valve leaflets, and elevated levels of these complexes have been linked to rapid progression from mild to severe stage [51]. However, the precise mechanisms by which they contribute to this accelerated progression remain unclear.

The interplay between lipids and inflammatory proteins in aortic stenosis is complex, extending beyond mere traditional lipid profiles. For example, while established risk factors like fibroblast growth factor-19 and interleukin-6 are increased in aortic stenosis, two-step Mendelian randomisation analyses have demonstrated a diminished causal role for these inflammatory proteins when assessed in conjunction with other lipid parameters, including triacylglycerols, sterols, and phosphatidylcholines [62]. These findings suggest that while these inflammatory proteins may be increased in patients with aortic stenosis, they may not be the direct drivers of the disease. Similarly, although apolipoprotein C-III-Lp(a) is associated with aortic stenosis, its contribution to the disease may be influenced by other interacting factors within the complex milieu of the aortic valve.

### 4.2. Extracellular Matrix Remodelling Factors

#### 4.2.1. Transforming Growth Factor-β1

Transforming growth factor-β1 (TGF-β1) is a multifunctional cytokine involved in regulating various cellular processes, including proliferation, differentiation, apoptosis, inflammation, and extracellular matrix remodelling. It plays a crucial role in cardiovascular health and has been implicated in heart diseases [63,64]. For example, in response to pressure overload, TGF-β1 activates connective tissue growth factor (CTGF) and endothelin, leading to cardiomyocyte hypertrophy and fibroblast proliferation [65,66]. These processes lead to overall cardiac remodelling.

Similar to its response to haemodynamic changes in cardiac remodelling, TGF-β1 may be implicated in aortic stenosis in the same mechanisms too. Elevated plasma TGF-β1 levels have been associated with aortic transvalvular gradients and left ventricular remodelling [67]. Altered sheer stress in aortic stenosis can activate TGF-β1 and promote fibrosis [68]. Recent research suggests that targeting TGF-β1 may be a promising therapeutic strategy for aortic stenosis, supported by the detection of increased TGF-β1 expression in human aortic stenosis biopsies [69,70].

#### 4.2.2. Matrix Metalloproteinases

Matrix metalloproteinases (MMPs) are key proteases involved in extracellular matrix degradation, one of the critical processes in aortic stenosis progression. By breaking down the extracellular matrix in the valve, MMPs contribute to valve thickening, stiffening, and ultimately, calcification [71]. Consequently, numerous studies have implicated MMPs in aortic valve remodelling and calcification, as summarised in Table 2. Notably, these studies consistently demonstrate elevated levels of multiple MMPs in patients with aortic stenosis compared to healthy individuals.

Increased MMPs may be attributed to a dysregulation between MMPs and their inhibitors, known as tissue inhibitors of metalloproteinases [72]. This imbalance can lead to excessive extracellular matrix degradation, resulting in the release of byproducts, such as elastin-derived peptides and integrins. These peptides exhibit chemotactic properties, stimulating valvular endothelial cell proliferation and an influx of calcium ions [73,74]. Moreover, these peptides may also contribute to angiogenesis within the valve by releasing angiogenic factors and facilitating the migration of valvular endothelial cells [75,76,77]. These combined effects ultimately contribute to the pathological hallmarks of aortic stenosis, which include distorted cusps, calcifications, and degeneration of structural components.

**Table 2:** MMP expression in aortic stenosis.

Study	Population	Detection (Sample)	Enzyme	Key Substrates	Plasma Levels in Median (Q1–Q3)	Key Findings
Lurins et al. [78]	55–80 years *n* = 18 (mild), 19 (moderate), 15 (severe), 50 (control)	ELISA (plasma)	MMP-1	Collagen I, II, III, VII, VIII and X	Exact values not shown	MMP-1 was lower in severe AS groups compared to mild or moderate AS.
			MMP-3	Fibronectin, laminin, gelatin I, III, IV and V, collagen III, IV, X and IX	Exact values not shown	No difference among the groups.
			MMP-9	Collagen IV and V, gelatin I and V	Exact values not shown	No difference among the groups.
Zhou et al. [79]	48–68 years *n* = 24 (mild), 31 (moderate), 24 (severe)	ELISA (plasma)	MMP-28	Casein	Mild = 0.74 (0.25–2.23) ng/mL Moderate = 1.46 (0.50–3.22) ng/mL Severe = 4.13 (1.54–6.18) ng/mL	MMP-28 was higher in severe AS than mild or moderate AS. MMP-28 correlated with increased pressure gradients
Matilla et al. [80]	43–81 years *n* = 112 (severe), 349 (control)	ELISA (plasma)	MMP-10	Fibronectin, gelatin I, III, IV and V, (weakly) collagen III, IV and V	Control = 593 (452–801) pg/mL Severe = 717 (552–1093) pg/mL	MMP-10 levels were elevated in patients with severe AS compared to controls and were correlated with TNF levels, suggesting a link to inflammation.
Shelbaya et al. [81]	ARIC study for tracking MI and CHD (1987–2019). 60 ± 6 years (*n* = 11,430) 76 ± 5 years (*n* = 4899) AS severity was determined using echocardiography	Olink Proteomics (plasma)	MMP-12	Elastin	Not applicable	Higher MMP-12 levels were linked to increased risk of incident AV hospitalisations.
Jian et al. [82]	*n* = 6 (AS), 6 (control) No AS severity determination was mentioned in the study	IHC, gel zymography (autopsy)	MMP-2	Gelatin I, collagen IV, V, VII and X	Not applicable (semi-qualitative measurements)	MMP-2 was associated with severe calcific aortic stenosis. The presence of MMP-2 in its pro form suggests a potential role in extracellular matrix deposition or healing.

Abbreviations: ARIC, atherosclerosis risk in communities; AS, aortic stenosis; AV, aortic valve; AVA, aortic valve area; ELISA, enzyme linked immunosorbent assay; IHC, immunohistochemistry; MMP, matrix metalloproteinase; PG, pressure gradient; TNF, Tumour necrosis factor; Vmax, peak aortic valve velocity.

However, it must be noted that the specific roles of each of the MMPs still remain largely unclear, as evidenced by conflicting findings in previous studies. For example, MMP-28 levels increase with disease severity, while MMP-1 levels decrease. While the dysregulation between MMPs and tissue inhibitors of metalloproteinases in aortic stenosis has been established [83], this equilibrium is still after all, a dynamic process in nature. Indeed, in the context of angiogenesis driven by extracellular matrix degradation, a balanced MMP-TIMP expression is essential for proper basement membrane assembly and endothelial cell function. Extrapolating this idea to aortic stenosis, it is then plausible that a similar balance might be restored in the advanced stages of the disease too. As such, the clinical usefulness of MMPs in staging aortic stenosis seems limited.

### 4.3. Immune Components

#### 4.3.1. Monocytes

Recent studies have highlighted the role of monocytes and macrophages in the progression of aortic stenosis. Monocytes infiltrate the aortic valve, subsequently becoming macrophages that take up oxidised lipids, contribute to inflammation and promote calcification of the aortic valve [84]. As such, higher monocyte counts have been associated with rapid aortic stenosis progression, with patients in the high monocyte group showing faster increases in aortic jet velocity and mean pressure gradient [85]. Patients with moderate to severe aortic stenosis exhibit increased numbers of CD14+ monocytes compared to controls, with an inverse correlation between monocyte count and aortic valve area [86]. Paradoxically, severe aortic stenosis has been linked to decreased circulating monocyte counts, suggesting a potential depletion as the disease progresses [87]. These findings indicate that monocytes/macrophages may contribute to aortic stenosis progression, potentially serving as biomarkers for disease severity.

As a common blood test in the management of aortic stenosis patients, monitoring monocyte count could provide valuable insights into disease severity. However, interpreting monocyte count requires caution due to the diverse biological roles of these cells and the potential influence of other comorbidities, such as diabetes mellitus and hypertension, which are commonly seen in aortic stenosis patients. Additionally, the role of monocytes in aortic stenosis may vary at different stages of the disease, even before and after surgical intervention [88,89]. Therefore, while monocyte count can be a useful biomarker, it is unlikely to be a stand-alone one. It should be considered in conjunction with other clinical and laboratory parameters.

#### 4.3.2. Neutrophil Extracellular Traps

Neutrophil extracellular traps (NETs) are extracellular, web-like structures formed by activated neutrophils. They consist of nuclear or mitochondrial chromatin decorated with various proteins, including citrullinated histone H3, neutrophil elastase, nucleosomes, myeloperoxidase, and others. While the primary role of NETs relates to combating infections by trapping microorganisms [90], their dysregulation has been implicated in immune-related cardiovascular diseases, including aortic stenosis [91]. Recent studies have demonstrated elevated levels of NET markers, including citrullinated histone H3 and nucleosomes, in both plasma and aortic valves of patients with severe aortic stenosis [92,93]. Notably, elevated levels of citrullinated histone H3 have been positively correlated with both interleukin-6 levels and the severity of aortic stenosis, as assessed by aortic valve area [93]. This observation suggests a potential positive feedback loop, where interleukin-6 promotes the formation of NETs, which in turn further enhances inflammation within the valve [94]. Moreover, NETs can activate macrophages, leading to the release of pro-inflammatory cytokines such as IL-1β and attracting further immune cells to the site [95]. Additionally, myeloperoxidase released by NETs can oxidise LDL particles, which then trigger an inflammatory response within the valve by facilitating more recruitment of immune cells [96,97]. Collectively, these findings suggest the involvement of NETs in disease progression through inflammatory pathways.

### 4.4. Glycoproteins

#### 4.4.1. Follistatin-like 1

Follistatin-like 1 (FSTL1), an extracellular glycoprotein primarily produced by mesenchymal cell lineage (cardiomyocytes, fibroblasts, adipocytes, and osteocytes), plays a crucial role in the development and maturation processes of aortic valves [98,99]. Given its critical role in valvular development, it is unsurprising that FSTL1 has emerged as a potential biomarker in patients with aortic stenosis. Indeed, FSTL1 expression has been shown to be increased in patients with this condition [99,100]. However, its precise role in the pathogenesis of aortic stenosis remains unclear.

While increased FSTL1 levels can potentially induce inflammatory responses in macrophages, contributing to disease progression [101], they have also been associated with reduced osteogenic differentiation of valvular interstitial cells [102]. This inhibition of osteogenesis may offer a potential protective mechanism against aortic valve calcification. Indeed, lower levels of serum FSTL1 have been associated with a higher risk of calcific aortic stenosis events and have been independently identified as predictors of calcific aortic valve disease, particularly in younger patients and those without established cardiovascular risk factors [103]. Furthermore, overexpression of FSTL1 has been shown to exert beneficial effects in other cardiovascular contexts, including reduced myocardial apoptosis, ischemia-reperfusion injury, and the risk of post-infarct myocardial rupture [103]. Taken together, these findings highlight the complex and potentially paradoxical role of FSTL1 in aortic stenosis, with both pro-inflammatory and protective effects. While FSTL1 undoubtedly influences various aspects of cardiovascular health, its overall impact on the development of aortic stenosis remains to be fully elucidated.

#### 4.4.2. Podoplanin

Podoplanin, an *O*-glycosylated transmembrane protein, is essential for the development of the heart. Notably, podoplanin expression is restricted to lymphatic endothelial cells and is absent in blood vessels, making it a valuable marker for lymphangiogenesis studies [104]. It has thus emerged as a promising biomarker for aortic stenosis. This is because in aortic stenosis, lymphangiogenesis is observed within the aortic valves, which are normally devoid of lymphatic vessels, to aid in the clearance of inflammatory debris and excess fluid [105]. This process is regulated by valvular myofibroblasts and mast cells [105]. Consistent with this understanding, podoplanin has been frequently localised around areas of calcification within aortic valves, where the differentiation of valvular interstitial cells into calcium-producing myofibroblast-like cells occurs [106].

The overall impact of podoplanin on aortic stenosis likely depends on the delicate balance between lymphangiogenesis and angiogenesis. While angiogenesis serves as the entry route for lipids and immune cells into the valve, lymphangiogenesis facilitates their clearance. An imbalance in favour of angiogenesis can lead to the accumulation of these factors within the aortic valve, contributing to inflammation and exacerbating calcification. This imbalance may be more pronounced in severely calcified valves compared to sclerotic valves, suggesting a potential role for podoplanin as a biomarker for severe aortic stenosis [105].

Studies so far have primarily focused on podoplanin expression within aortic valve tissue, with limited data available on circulating plasma levels of podoplanin. Furthermore, its expression is not limited to the heart and can be elevated in other conditions, such as cancer [107,108]. These factors may limit the specificity and clinical utility of plasma podoplanin as a biomarker for aortic stenosis and warrant further investigation.

### 4.5. Others

#### 4.5.1. Trimethylamine N-Oxide

The emerging field of microbiota-derived metabolites has garnered significant interest in recent years, with implications for various seemingly unrelated health conditions, including aortic stenosis. In particular, trimethylamine N-oxide (TMAO) has been implicated in the pathogenesis of aortic stenosis [109,110]. TMAO may contribute to aortic stenosis by promoting osteogenic differentiation of valvular interstitial cells, potentially through mechanisms involving oxidative stress activation and endoplasmic reticulum and mitochondrial dysfunction [111]. This process may involve the activation of inflammatory signalling cascades, such as mitogen-activated protein kinase and nuclear factor-κB, leading to the recruitment of leukocytes to the valve [112]. Consistent with the mechanisms, patients with elevated TMAO levels have demonstrated poorer outcomes after transcatheter aortic valve replacement (TAVR), including an increased risk of all-cause mortality, prosthetic valve thrombosis, and stroke [110,113]. These findings suggest that TMAO may not only contribute to the development of aortic stenosis but also influence its clinical course and prognosis.

#### 4.5.2. Sestrin-2

Sestrins are stress-inducible proteins with antioxidant and anti-inflammatory properties, regulating key cellular processes including tissue growth and metabolism [114]. Among the sestrin family, sestrin-2 has been extensively studied in aortic stenosis due to its prominent role in oxidative stress responses [115,116]. Sestrin-2 primarily plays a protective role in aortic stenosis by regulating reactive oxygen species production and preventing the oxidation of LDL [117]. It may also play a role in regulating macrophage polarisation, potentially shifting pro-inflammatory M1 macrophages towards a less inflammatory M2 phenotype [118,119,120]. This shift could then help mitigate inflammation and tissue damage within the aortic valve. Indeed, silencing its gene has been shown to exacerbate M1 macrophage-driven inflammation, further supporting its role in regulating macrophage phenotype [118]. Together, the suppression of inflammation and oxidative stress by sestrin-2 may contribute to the attenuation of valve calcification [120].

The clinical utility of sestrin-2 as a biomarker for aortic stenosis remains uncertain, as current research primarily relies on pre-clinical models. While elevated plasma sestrin-2 levels have been observed in other cardiovascular conditions [121,122,123], increased sestrin-2 protein expression has been observed in aortic stenosis valves ex vivo [120]. This suggests a potential feedback mechanism regulating oxidative stress and inflammation within the valve. Given its role in immunoregulation, particularly macrophage polarisation (discussed in Section 4.3.1), sestrin-2 may offer a promising therapeutic target.

#### 4.5.3. Extracellular Vesicles

Extracellular vesicles, including exosomes and microvesicles, are small lipid-encapsulated particles released by cells to facilitate intercellular communication by carrying various biomolecules, such as nucleic acids, lipids, and proteins [124]. Studies have shown elevated levels of circulating extracellular vesicles in patients with severe aortic stenosis, with a significant decrease observed following valve intervention [125]. While extracellular vesicles themselves may not have intrinsic pro-coagulant properties, they can trigger pro-coagulant activity in cardiac microvascular endothelial cells, potentially contributing to thromboembolic events. Intriguingly, extracellular vesicles trapped in valves and blood vessel walls can contribute to calcification [126]. Notably, tissue-specific extracellular vesicles-derived molecules have been shown to modulate calcification in valvular interstitial cells and smooth muscle cells [127]. These findings suggest that extracellular vesicles may play a significant role in the pathogenesis of aortic stenosis via promoting calcification. However, further research is warranted to fully elucidate their specific mechanisms of action and to explore their clinical translation as potential biomarkers for this condition.

## 5. Practical Considerations for Clinical Use

### 5.1. Current Clinical Applications of Biomarkers

Despite the emergence of novel biomarkers, their clinical utility remains limited due to several challenges, including conflicting results across studies, the influence of ethnicity on biomarker levels, and the lack of standardised cut-off thresholds and measurement methods. These factors hinder their widespread adoption in clinical practice. The complex interplay between various biomarkers and their potential synergistic or antagonistic effects also requires further investigation. Current research efforts are focused on understanding the specific mechanisms by which these biomarkers contribute to aortic stenosis (Table 3).

Based on our analysis in Table 3, Lp(a) appears to be the most promising biomarker for aortic stenosis stratification. Its significant association with faster rates of hemodynamic progression has attracted considerable attention from both academic and pharmaceutical sectors. Several companies have invested in research and development to target Lp(a) as a therapeutic target. The growing recognition of Lp(a)’s importance is already reflected in its inclusion in various clinical guidelines and consensus statements for other cardiovascular disease, such as the Lipoprotein(a) Consensus 2022 by the European Atherosclerosis Society [128] and the 2021 Guidelines for the Management of Dyslipidaemia for the Prevention of Cardiovascular Disease in Adults issued by the Canadian Cardiovascular Society [129].

While Lp(a) has not been explicitly integrated into aortic stenosis guidelines, the 2010 EAS consensus statement for ASCVD [130] may provide a framework for its use in risk stratification. This statement suggests using an Lp(a) threshold of 50 mg/dL as a ‘risk enhancer’ to refine individual risk assessments [131]. However, it is important to note that the Lp(a) threshold for aortic stenosis appears to be higher. A recent study suggests that Lp(a) levels exceeding 100 mg/dL are associated with severe aortic stenosis and the need for surgical interventions, regardless of initial disease severity [132].

### 5.2. Variability in Biomarker Measurement

Significant challenges remain in translating the biomarkers discussed in this review into routine clinical diagnostics for aortic stenosis, notably issues of reproducibility, standardisation, and cost-effectiveness. The ability to track changes in biomarker levels over time is crucial not only for optimising individual patient management but also for conducting robust epidemiological studies aimed at understanding the role of these biomarkers in aortic stenosis, which is still largely unclear currently. In the following sections, we offer our perspectives on the challenges that need to be addressed to realise their full potential.

#### 5.2.1. Assay Standardisation and Reproducibility

Let’s use Lp(a) as an example to illustrate this point. Despite the growing recognition of Lp(a) as a crucial cardiovascular risk factor and its endorsement in established guidelines, a significant gap remains in the standardisation of its measurement. While clinical laboratories commonly employ immunoassays for Lp(a) quantification, the inherent size heterogeneity of Lp(a) particles, stemming from variations in the apolipoprotein(a) component, poses a considerable challenge. This heterogeneity can lead to discrepancies in results obtained from different assays, hindering accurate comparisons and potentially impacting clinical decision-making. Although mass concentration (mg/dL) is the most frequently reported unit, the lack of a universally accepted conversion factor to particle number (nmol/L), due to this size variability, further complicates the interpretation of Lp(a) levels. This absence of a ‘gold standard’ assay and the ongoing efforts towards standardisation underscore the need for continued research and development to improve the accuracy and reliability of Lp(a) measurement, ensuring its effective translation into clinical practice.

#### 5.2.2. Economic Implications of Biomarker Use

The cost-effectiveness of incorporating novel biomarkers into the clinical management of aortic stenosis is a complex issue with no simple answer. While biomarkers offer the potential for more efficient resource allocation, their actual cost-effectiveness hinges on a multitude of factors. A key consideration is the cost of the biomarker assay itself; an expensive test could negate any potential savings from reduced utilisation of other diagnostic modalities. Furthermore, the accuracy and reliability of the biomarker are important. A highly accurate biomarker that effectively identifies low-risk individuals, thereby reducing the need for frequent echocardiographic follow-up, is more likely to be cost-effective. Conversely, a less accurate biomarker could lead to unnecessary further testing or, more concerningly, miss high-risk patients, potentially increasing overall healthcare costs due to the expenses associated with managing disease progression and complications. Beyond the direct cost of the assay, implementation costs, including equipment, training, and protocol development within clinical laboratories, must also be factored into the equation.

Therefore, while biomarkers hold promise for improving the efficiency and potentially reducing the overall cost of aortic stenosis management, we think that rigorous cost-effectiveness analyses, taking into account all relevant variables discussed above, are essential to determine their true value and guide clinical implementation. It cannot be assumed that biomarkers will automatically be more cost-effective; each biomarker and its intended clinical use case must be evaluated carefully.

#### 5.2.3. Genetic Influences on Biomarker Levels

The influence of genetic background also represents a significant consideration. Just as genetic variations can impact the levels of biomarkers in other cardiovascular conditions, they may also play a role in influencing biomarker levels in aortic stenosis (see Section 4.1.1 for example). Individuals of different ethnicities, the genetic basis of Lp(a) levels, may exhibit varying risk thresholds. For instance, individuals of Chinese, South Asian, and Black African descent tend to have higher Lp(a) levels compared to White individuals [133]. This highlights the importance of considering ethnicity when interpreting Lp(a) levels and making clinical decisions. A patient with a genetic predisposition to higher levels of a particular biomarker might exhibit elevated levels even with mild aortic stenosis, potentially leading to overdiagnosis or unnecessary interventions. Conversely, another individual with a genetic predisposition to lower levels might have deceptively normal or only mildly elevated biomarker levels despite having more advanced disease. Therefore, when evaluating biomarkers for aortic stenosis diagnosis, it is crucial to acknowledge the potential confounding effects of genetic background.

As such, we think that future research exploring the interplay between genetics and biomarker levels in aortic stenosis is essential to refine diagnostic strategies, improve risk stratification, and personalise treatment approaches. Integrating genetic information alongside biomarker data holds the promise of enhancing the accuracy and reliability of aortic stenosis diagnosis, leading to more informed clinical decision-making.

#### 5.2.4. Limitations of Current Evidence

A key weakness of many current studies included in this review is their observational design, which limits the ability to draw definitive conclusions about causality. Observational studies are susceptible to various biases that can confound the interpretation of results. Selection bias, for example, could arise if the study population is not representative of the broader population of individuals with or at risk for aortic stenosis. Confounding factors, such as age, comorbidities, and other lifestyle factors, may also influence both biomarker levels and the presence or progression of aortic stenosis, potentially masking or exaggerating the true association between the two. We agree that larger, multi-centre validation studies are essential to address these limitations. Such studies, with their increased statistical power and diverse patient populations, will be crucial for confirming the observed associations, minimising the impact of potential biases, and establishing the true clinical utility of these biomarkers in the diagnosis, prognosis, and management of aortic stenosis. Future research needs to prioritise the design and implementation of such studies to rigorously evaluate the performance of these biomarkers and ultimately translate their potential into improved patient care.

### 5.3. Areas for Future Investigation

While echocardiography remains the gold standard for aortic stenosis diagnosis, the integration of biomarkers with imaging findings holds promise for enhancing risk stratification and personalising patient management. For example, patients with elevated levels of growth/differentiation factor 15, soluble suppression of tumorigenicity 2, and NT-pro-BNP may benefit from earlier consideration for aortic valve replacement compared to those with no elevated biomarkers [134]. Combining echocardiography with plasma biomarkers may therefore provide a more individualised risk assessment for patients with aortic stenosis. However, the precise role of biomarkers in complementing echocardiographic findings is still unknown and being investigated. It is important to note that none of the biomarkers discussed in this review have been formally adopted for clinical risk stratification.

Therefore, we think the future direction of research should look into how biomarkers can aid in enhancing imaging diagnosis and practically the cost-effectiveness of multi-modal assessment and establish standardised protocols for integrating biomarkers into routine clinical practice. Specifically, here are some key questions we should look into in future research on whether or not biomarkers can: (1) improve risk stratification—even in patients with mild aortic stenosis on echocardiography, biomarkers may help identify those at higher risk of rapid progression or adverse events; (2) refine treatment decisions—biomarkers may provide additional information to guide decisions about the timing of aortic valve replacement, particularly in patients who are asymptomatic or have borderline echocardiographic findings; (3) enhance monitoring of treatment response—biomarkers could potentially be used to monitor the effectiveness of medical therapies; and (4) identify early disease—biomarkers may be able to detect subtle changes associated with aortic stenosis development before they are apparent on echocardiography, offering the possibility of earlier intervention.

Lastly, the era of precision medicine has highlighted the importance of personalised approaches to healthcare. Traditional ‘one-size-fits-all’ clinical trials often fail to account for the significant heterogeneity among patients. To address this, patient-centred trial designs, such as basket, umbrella, and platform trials, have emerged [135]. While Lp(a) seems to show promise as a potential biomarker, it is important to recognise that individual responses to disease progression may vary. Therefore, a personalised approach that considers patient-specific factors, such as family history, genetic variations, lifestyle, and comorbidities, is essential in prediction strategies.

## 6. Conclusions

In conclusion, while echocardiography remains a crucial diagnostic tool for the haemodynamic and anatomical assessment of aortic stenosis, emerging biomarkers offer the potential to enhance risk stratification and guide treatment decisions in adjunct to imaging modalities. While BNP has been incorporated into guidelines, further research is needed to validate the clinical utility of other promising biomarkers such as Lp(a), FSTL1, and inflammatory markers. A multi-modality approach that considers a combination of clinical, imaging, and biochemical biomarkers may ultimately provide a more accurate and personalized assessment of patient risk. Future studies should focus on identifying the optimal combination of biomarkers and developing strategies to translate these findings into clinical practice.

## Figures and Tables

**Table 1:** Clinical trials * targeting Lp(a) gene expression in subjects with or at risk of aortic stenosis.

Study or Drug Name; Identifier; Status	Phase	Recruitment	Intervention	Mechanism of Action	Primary Outcome
OCEAN(a); NCT05581303; Active, not recruiting	3	7297 subjects 18–85 years Lp(a) ≥ 200 nmol/L History of AS At least one additional risk factor	Olpasiran; SC injection of 10, 75, or 225 mg every 12 weeks vs. placebo	siRNA that reduces Lp(a) synthesis in the liver	Time to first occurrence of CVD death, myocardial infarction, or urgent coronary revascularisation [56].
Zerlasiran; NCT05537571; completed	2	180 subjects 18–80 years Lp(a) ≥ 150 nmol/L At high risk of AS	Zerlasiran (SLN360); SC single-dose injection of 30, 100, 300, or 600 mg vs. placebo	Double-stranded siRNA that targets *LPA* gene mRNA	Change in Lp(a) plasma levels from baseline [57].
KRAKEN; NCT05563246; completed	2	233 subjects 40 years above Lp(a) ≥ 175 nmol/L At high risk of AS BMI 18.5–40 kg/m^2^	Muvalaplin (LY3473329); daily oral for 12 weeks (dose unknown) vs. placebo	siRNA that targets hepatic *LPA* gene	Change in Lp(a) plasma levels from baseline [58].

* We excluded trials that did not recruit aortic stenosis subjects, including Lp(a)HORIZON, ORION-11, etc. Abbreviations: AS, aortic stenosis; BMI; body mass index; CVD; cardiovascular disease; Lp(a), lipoprotein(a); *LPA*, lipoprotein(a) gene; mRNA, messenger RNA; SC, subcutaneous; siRNA, small interfering RNA; vs., versus.

**Table 3:** Summary of circulating biomarkers for aortic stenosis.

Biomarker	Mechanism of Action	Plasma Levels in Aortic Stenosis	Limitations in Current Understanding	Limitations in Clinical Practicality
Lp(a) conjugated with oxidised phospholipid	Promotes valvular calcification and inflammation via the ATX-LPA pathways.	Positively correlated with disease progression, but may decrease as the disease progresses.	Ethnic/racial disparities	**Limited clinical guidelines:** current guidelines do not routinely recommend Lp(a) testing. **Cost and availability:** Lp(a) testing can be more expensive than standard lipid panels and may not be readily available in all clinical settings. **Lack of treatment options:** there are currently no treatment options specifically targeting Lp(a).
Lp(a) conjugated with apolipoprotein C-III	Induces mitochondrial dysfunction to initiate calcification.	Elevated levels in patients with mild aortic stenosis may indicate a higher risk of rapid progression.	Limited cohort studies	
Transforming growth factor-β1	Promotes fibrosis in valvular endothelial cells and interstitial cells.	Elevated levels may indicate left ventricular remodelling.	Primarily based on animal studies	**Limited data:** its use as a routine diagnostic biomarker is not yet established. **Variability in assay methods:** the sensitivity and specificity of assays can vary depending on the assay methodology.
MMP-1	Degrades extracellular matrix leading to valve thickening.	Increased levels in subclinical patients may indicate a higher risk of progression.	Limited cohort studies	**Complexity and cost:** measuring specific MMPs often requires specialised assays, which can be complex and expensive. **Impracticality:** many different MMPs are involved, and measuring all of them would be impractical and costly for routine clinical use. **Lack of clinical guidelines:** there are currently no established clinical guidelines recommending routine MMP testing for the diagnosis or management of aortic stenosis. **Limited clinical utility:** there are currently no established clinical guidelines recommending routine MMP testing for the diagnosis or management of aortic stenosis.
MMP-2	Unknown	Inconsistent findings due to variable expression patterns in stenotic valves.	Studies have been done on biopsies, not plasma	
MMP-3	Unknown	Levels not different between disease stages.	Lack of studies	
MMP-9	Unknown	Levels not different among disease stages.	Lack of studies	
MMP-10	Contributes to valve extracellular matrix degradation.	Patients with elevated levels may be at higher risk for rapid disease progression to severe stages.	Limited understanding of its expression in early to moderate stages	
MMP-12	Degrades elastin contributing to valve stiffening.	Increased levels in patients with early or mild aortic stenosis indicate higher risk for progression to moderate or severe stages.	Pathophysiological mechanisms not clear	
MMP-28	Unknown	Increased plasma levels indicate an increasing risk for progression.	Causal role not clear	
Neutrophil extracellular trap	Associated with valve immune responses.	Elevated plasma levels may be associated with an increased risk of progression to severe stages.	Mechanisms not clear	**Technical challenges:** NET formation is a complex process, and accurately measuring it in clinical samples presents significant technical challenges. Current detection methods such as flow cytometry are often time-consuming. **Lack of standardisation:** this makes it difficult to compare results across different laboratories and interpret findings consistently.
Follistatin-like 1	Complex role; may have both beneficial and detrimental roles	Lower levels have been associated with a higher risk of valve calcification.	Mechanisms not clear	**Lack of standardised assays:** reliable and widely available assays may not be accessible in most clinical settings. **Emerging research area:** its role in aortic stenosis is an area of ongoing research.
Monocytes	Contribute to inflammation and calcification.	Elevated counts associated with rapid disease progression, but decreased in severe stages.	Roles and mechanisms at different disease stages are not known.	**Limited specificity:** elevated monocyte counts can be observed in various inflammatory conditions, not just aortic stenosis. This lack of specificity limits their diagnostic value for aortic stenosis alone. **Focus on cell count:** routine CBCs typically provide a total monocyte count, not their functional status (e.g., their activation state, inflammatory phenotype, etc.).
Trimethylamine N-oxide	Promotes osteogenic differentiation of valvular interstitial cells.	Higher levels seen in patients with moderate-severe stages.	Causal relationship not clear	**Complexity and impracticality:** it typically needs specialised laboratory equipment and techniques, such as liquid chromatography-mass spectrometry, which may not be readily available in all clinical laboratories. **Limited clinical utility:** TMAO testing is currently considered more of a research tool rather than a routine clinical test.
Sestrin-2	Protective role in shifting macrophage polarisation from M1 to M2.	Higher levels are seen in calcific aortic valve disease, and it may delay progression of disease.	Lack of cohort studies.	**Limited availability:** assays may not be readily available in most clinical laboratories. **Research focus:** most research has been conducted in pre-clinical models or has focused on limited patient cohorts.
Extracellular vesicles	Triggers procoagulant activity in cardiac microvascular endothelial cells.	Elevated levels in severe aortic stenosis.	Carry non-specific factors	**Sensitivity and specificity:** developing sensitive and specific methods for detecting and quantifying EV-associated biomarkers (e.g., proteins, nucleic acids) is an ongoing area of research. **Cost-effectiveness:** analysis can be expensive and time-consuming, potentially limiting its cost-effectiveness in routine clinical settings.
Podoplanin	Complex role; influenced by angiogenesis and lymphangiogenesis.	More pronounced expression in severely calcified valves compared to early-stage sclerotic valves.	Not heart-specific; may be elevated in other conditions. Limited plasma data.	**Limited availability:** assays may not be readily available in most clinical laboratories. **Invasive nature:** Valve tissue analysis would likely be the most direct way to assess podoplanin expression in the context of aortic stenosis. This invasive nature limits the practicality of routinely assessing podoplanin expression for diagnostic purposes.

Abbreviations: ATX, autotaxin; EV, extracellular vesicles; Lp(a), lipoprotein(a); MMP, matrix metalloproteinase; NET, neutrophil extracellular trap; TMAO, trimethylamine N-oxide.
